# Frontal Meningoencephalocele With Cerebrospinal Fluid Rhinorrhea: A Case Report

**DOI:** 10.7759/cureus.46343

**Published:** 2023-10-02

**Authors:** Abdulaziz Y AlEnezi, Mahdi Aljawad, Ammar W Baltoyour, Shahad A Alotaibi, Mohammad S Alzahri

**Affiliations:** 1 General Practice, Arabian Gulf University, Manama, BHR; 2 Radiology, Qatif Central Hospital, Qatif, SAU; 3 General Practice, Dhahran Eye Specialist Hospital, Dhahran, SAU; 4 College of Medicine, Sulaiman Al Rajhi University, Al Bukayriyah, SAU; 5 Emergency Medicine, Dallah Hospital, Riyadh, SAU

**Keywords:** case report, magnetic resonance imaging, computed tomography, meningoencephalocele, cerebrospinal fluid rhinorrhea, traumatic brain injury

## Abstract

Traumatic brain injuries are a significant public health concern often associated with immediate consequences. However, delayed complications can manifest, including rare congenital neural tube defects such as encephaloceles. We present a case of a 45-year-old male with a history of traumatic brain injuries who developed a posttraumatic frontal meningoencephalocele associated with cerebrospinal fluid rhinorrhea. This case emphasizes the need for vigilance in assessing patients with a history of head trauma for delayed complications, even years after the initial injury. Early diagnosis and intervention can significantly impact outcomes.

## Introduction

Traumatic brain injuries are a significant public health concern, often resulting from various forms of accidents, including motor vehicle collisions. While the immediate consequences of traumatic brain injuries can be life-threatening, long-term complications may also manifest, requiring ongoing medical attention [1]. We present a case of a 45-year-old male who suffered a traumatic brain injury several years ago, leading to a subsequent and rare complication of a posttraumatic frontal meningoencephalocele associated with cerebrospinal fluid rhinorrhea. Traumatic brain injuries, characterized by disruptions in normal brain function, are frequently associated with skull fractures [2]. The presentation of rhinorrhea indicated a potential communication between the intracranial and nasal cavities, necessitating a thorough investigation [1,2]. This case underscores the importance of vigilant follow-up and the recognition of delayed complications following traumatic brain injuries, as meningoencephaloceles and cerebrospinal fluid rhinorrhea are uncommon sequelae of head trauma. Understanding the various clinical signs and symptoms associated with meningoencephaloceles in patients with a history of traumatic brain injuries is crucial for early diagnosis and intervention.

## Case presentation

A 45-year-old male patient, with a history of a motor vehicle accident five years ago, presented to our neurosurgery clinic with complaints of clear fluid drainage from his right nostril, particularly when bending forward or straining, and a gradually enlarging soft swelling in the right frontal region. The patient reported that these symptoms had been present for approximately six months and were progressively worsening. Despite the persistence of symptoms, he denied experiencing any neurological deficits such as headaches, seizures, or changes in mental status. Detailed history and physical examination were performed, followed by a comprehensive work-up, including laboratory investigations and imaging studies.

On physical examination, the patient appeared well-nourished and in no acute distress. Neurological examination did not reveal any focal deficits. A nasal endoscopy was performed, confirming the presence of clear fluid leakage from the right nostril upon the application of gentle pressure to the swelling.

The differential diagnosis for the patient's presentation included rhinorrhea of cerebrospinal fluid secondary to a traumatic meningoencephalocele, intranasal mass lesions, sinonasal tumors, and infectious etiologies. Given the patient's history of a motor vehicle accident and the imaging findings, a traumatic meningoencephalocele was considered the most likely diagnosis.

Laboratory investigations, including a complete blood count and basic metabolic panel, were within normal limits. Cerebrospinal fluid analysis obtained from the nasal discharge confirmed the presence of beta-2-transferrin, consistent with rhinorrhea of cerebrospinal fluid.

**Figure 1:**
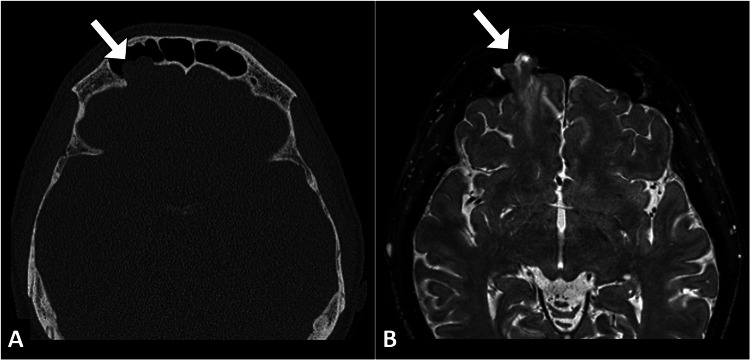
Axial CT head image with a bone window (A) revealing a notable bone defect (arrow) in the right anterior cranial fossa. The corresponding T2-weighted MR image (B) demonstrates herniation of brain tissue through this defect (arrow), consistent with a frontal meningoencephalocele. CT: computed tomography; MR: magnetic resonance

Imaging studies were crucial for further evaluation. Computed tomography and magnetic resonance imaging of the brain and skull were obtained, which revealed a bony defect in the anterior cranial fossa with herniation of the meninges and brain parenchyma, indicative of a posttraumatic frontal meningoencephalocele (Figure 1).

Based on the history, physical examination, cerebrospinal fluid analysis, and imaging studies, the patient was diagnosed with a posttraumatic frontal meningoencephalocele, resulting in rhinorrhea of cerebrospinal fluid.

The patient's management involved a multidisciplinary approach with neurosurgery and otolaryngology teams. Surgical intervention was recommended to repair the meningoencephalocele and close the cranial defect. The patient consented to the surgical procedure, which was scheduled for the following week. During the procedure, the frontal bone defect was repaired using an autologous bone graft, and the herniated meninges and brain tissue were carefully repositioned into the cranial vault. The dura was then closed in a watertight fashion. The patient had an uneventful postoperative course with no complications.

The patient was closely monitored in the postoperative period for any signs of infection or cerebrospinal fluid leakage. He was kept in a semi-recumbent position to minimize intracranial pressure. Intravenous antibiotics were administered prophylactically. No signs of infection or cerebrospinal fluid leakage were observed, and the patient's neurological status remained stable throughout his hospital stay.

The patient was discharged on postoperative day five with instructions for strict bed rest and head elevation for the next two weeks. He was advised to avoid strenuous activities and heavy lifting for six weeks. Follow-up appointments were scheduled at regular intervals to monitor his progress and ensure the resolution of rhinorrhea of cerebrospinal fluid and the absence of complications.

## Discussion

The presented case sheds light on the rare and intriguing condition of traumatic meningoencephalocele, a subtype of congenital neural tube defects. Unlike typical encephaloceles that result from insufficient skull fusion during fetal development, traumatic meningoencephaloceles are associated with head trauma [1].

Traumatic meningoencephaloceles occur due to the disruption of the skull and meningeal layers as a consequence of severe head injury [1]. These traumatic events create an opening through which brain tissue, meninges, and cerebrospinal fluid can herniate. While such cases are relatively rare, they demand careful consideration in the context of head trauma, as early diagnosis and intervention are critical for favorable outcomes [1,3].

The clinical presentation of traumatic meningoencephaloceles can vary significantly depending on the size and location of the herniation. Patients may exhibit a range of symptoms, including clear fluid rhinorrhea, headaches, seizures, and even focal neurological deficits. The presentation in our case, characterized by clear fluid drainage from the right nostril and a frontal soft swelling, was indicative of a potential traumatic meningoencephalocele [4].

Diagnosing traumatic meningoencephaloceles requires a systematic approach that includes thorough physical examinations, imaging studies, and laboratory investigations [5]. In our case, imaging studies, including computed tomography and magnetic resonance imaging, played a pivotal role in confirming the diagnosis by revealing a bony defect in the anterior cranial fossa with herniated meninges and brain parenchyma [4].

Management of traumatic meningoencephaloceles typically necessitates surgical intervention [4,5]. The primary goal of surgery is to repair the cranial defect, reposition the herniated brain tissue, and ensure a watertight closure of the dura mater. In our case, the patient underwent a successful surgical procedure involving the repair of the frontal bone defect using an autologous bone graft and the careful repositioning of herniated tissues. The postoperative period was uneventful, highlighting the importance of prompt surgical intervention and close monitoring [2].

## Conclusions

This case of traumatic meningoencephalocele highlights the distinct challenges posed by neural tube defects resulting from head trauma. It underscores the importance of vigilance in assessing patients with a history of head trauma, even years after the initial injury, as early diagnosis and multidisciplinary intervention are crucial. The case underscores the significance of prompt surgical management and close monitoring to achieve positive patient outcomes. Continued research into the long-term outcomes and risk factors associated with traumatic meningoencephaloceles will enhance our understanding of this condition and improve patient care in cases of head trauma-related complications, offering hope for improved quality of life for affected individuals.
